# Numerical Modeling of Silicon Photodiodes for High-Accuracy Applications Part II. Interpreting Oxide-Bias Experiments

**DOI:** 10.6028/jres.096.024

**Published:** 1991

**Authors:** Jon Geist, Rainer Köhler, Roland Goebel, A. M. Robinson, C. R. James

**Affiliations:** National Institute of Standards and Technology, Gaithersburg, MD 20899; BIPM, F-92310 Sèvres, France; University of Alberta, Alberta, Canada T6G 2G7

**Keywords:** induced junction photodiode, inversion layer photodiode, numerical modeling, numerical simulation, oxide-bias experiment, quantum efficiency

## Abstract

The semiconductor device modeling program PC-1D and the programs that support its use in high-accuracy modeling of photodiodes, all of which were described in Part I of this series of papers, are used to simulate oxide-bias self-calibration experiments on three different types of silicon photodiodes. It is shown that these simulations can be used to determine photodiode characteristics, including the internal quantum efficiency for the different types of photodiodes. In the latter case, the simulations provide more accurate values than can be determined by using the conventional data reduction procedure, and an uncertainty estimate can be derived. Finally, it is shown that 0.9997 ± 0.0003 is a nominal value for the internal quantum efficiency of one type of photodiode over the 440 to 460 nm spectral region.

## 1. Introduction

Part II of this series of papers presents the results of high-accuracy simulations of various oxide-bias experiments performed on silicon photodiodes. The simulations were carried out by using the semiconductor device modeling program PC-1D^1^ and a set of three programs designed to support the use of PC-1D in this specific application. Both PC-1D and the support programs are described in Part I of this series of papers.

The oxide-bias experiment [1–3] consists of recording the ratio of the photocurrent as a function of oxide-bias voltage to the zero-bias photocurrent during irradiation of the photodiode with a stable photon flux. The oxide bias is usually applied with a transparent water-drop electrode. A review is given in reference [3].

Part II is organized as follows: Section 2 describes the simulation of an oxide-bias, self-calibration experiment on an EG&G UV444B type photodiode. The purpose of this simulation is to demonstrate the derivation of an accurate value for the zero-bias quantum efficiency including an uncertainty estimate. Section 3 describes the simulation of an oxide-bias experiment on a Hamamatsu 1337 type photodiode. The purpose of this simulation is to determine nominal values for the charge trapped in the oxide covering the front surface of the photodiode, and for the surface recombination velocity at the oxide-silicon interface. Section 4 describes the simulation of an oxide-bias experiment on a UDT UV100 type photodiode. The purpose of this simulation is to determine a nominal high-accuracy value for the internal quantum efficiency of this type of photodiode and the nominal wavelength region where this value is valid. Section 5 gives the conclusions of Part II.

## 2. EG&G UV444B Photodiode

**Figure 1:**
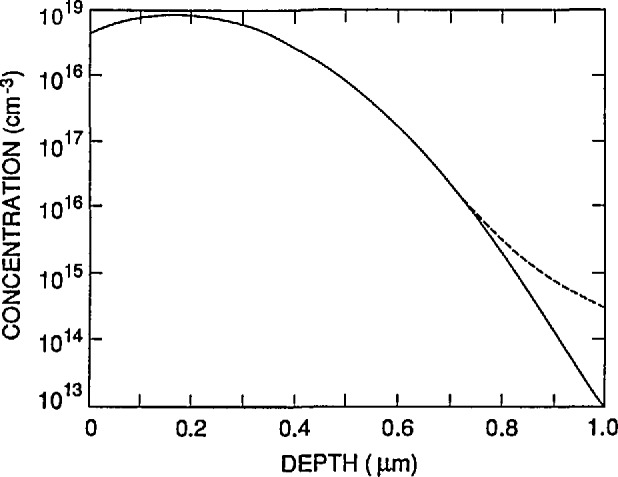
The front-region boron concentration (continuous line) and the equilibrium hole concentration (dashed line) calculated by PC-1D for a typical EG&G UV444B type photodiode.

The EG&G UV444B silicon photodiode was the first of its type to be self-calibrated by using the oxide-bias experiment [2]. This type of photodiode consists of a 300 µm thick, 300 Ω cm, phosphorus-doped, *n*-type silicon substrate with an *n*^+^-type phosphorus diffusion in the rear surface, a *p*^+^-type boron diffusion in the front surface, and a nominally 120 nm thick, thermally grown oxide-passivation layer over the front surface. Figure 1 shows the front-region doping profile that was obtained from spreading resistance measurements on a representative UV444B photodiode. Figure 1 also shows the equilibrium hole concentration calculated for this doping distribution by using PC-1D.

The rear-region, *n*^+^-type diffusion in the UV444B was not modeled; instead it was assumed that the recombination velocity at the rear of the device was zero. This assumption is not physically realizable, but it results in a mirror for minority carriers at the rear of the device, just as does the *n*^+^-type diffusion. This assumption is useful because it requires fewer finite elements, thereby leaving more of them available for modeling the front region and depletion region.

**Figure 2:**
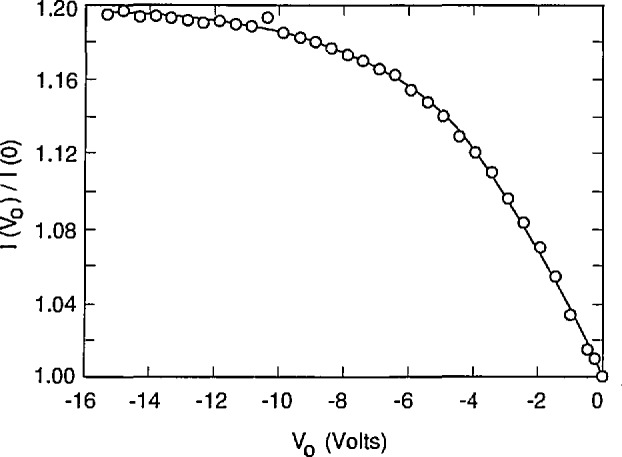
Ratio of the photocurrent *I*(*V*_0_) as a function of oxide-bias voltage *V*_0_ to the photocurrent *I*(0) at zero-bias voltage as measured on an EG&G UV444B silicon photodiode and as calculated with PC-1D with a front-surface recombination velocity *S* = 6000 cm/s and an oxide trapped charge number density *N*_ss_ = 1.942 × 10^12^ cm^−2^.

Figure 2 compares simulated and experimental results of an oxide-bias experiment on a UV444B photodiode for irradiation by a 3 mm diameter spot of 476.2 nm laser radiation through a water-drop electrode. Reference [4] describes the apparatus used to obtain the experimental data. The absorption-coefficient data in the file SIL_WEAK.ABS used for this simulation were calculated from eq [5] that was fit to the data measured by Weakliem and Redfield [6]. These data are described in Part I. Both the surface recombination velocity *S* and the number density *N*_ss_ of the oxide fixed charge were adjusted in the simulation to obtain a good fit to the experimental data in figure 2.

The procedure used to fit the simulated data to the experimental data shown in figure 2 was as follows: first, a value of *S* was chosen, then the value of *N*_ss_ was adjusted until the average residual of the fit to the photocurrent ratio was zero for the last two data points in the figure. This gave a value of *N*_ss_ for each value of *S* that was chosen. The best combination of *S* and *N*_ss_ was then obtained by minimizing the residual standard deviation over all of the data shown in figure 2.

**Figure 3:**
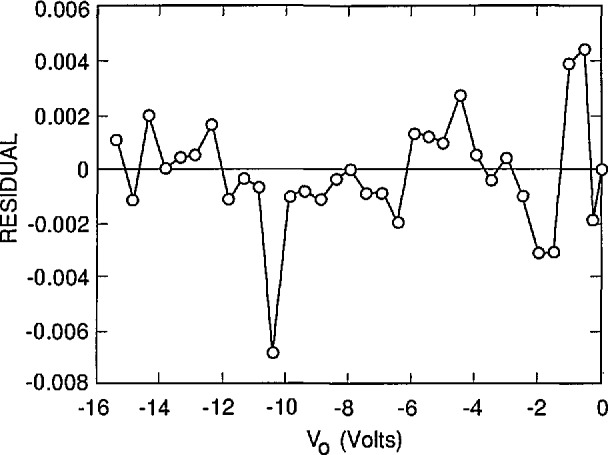
The residuals between the experimental data of figure 2 and the simulated data calculated by PC-1D when *S* = 6000 cm/s and *N*_ss_ = 1.942 × 10^12^ cm^−2^.

**Figure 4:**
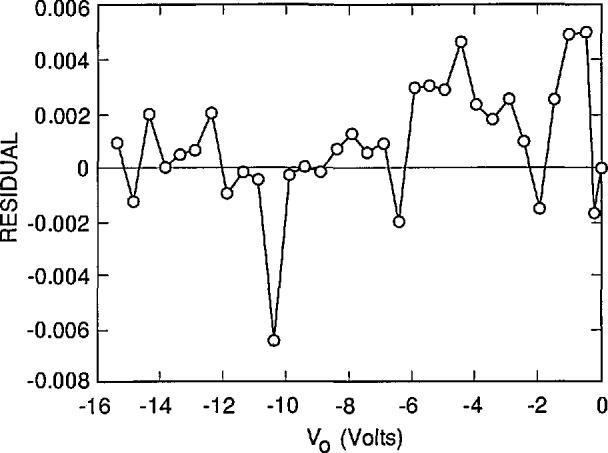
The residuals between the experimental data of figure 2 and the simulated data calculated by PC-1D when *S* = 5000 cm/s and *N*_ss_ = 2.011 × 10^12^ cm^−2^.

**Figure 5:**
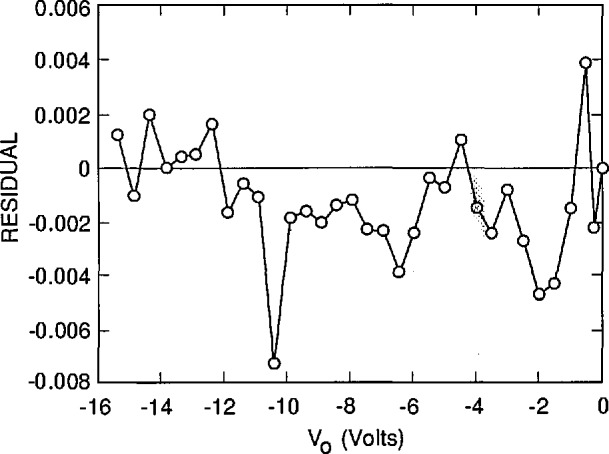
The rcsiduals between the experimental data of figure 2 and the simulated data calculated by PC-1D when *S* = 7000 cm/s and *N*_ss_ = 1.883 × 10^12^ cm^−2^.

Figures 3, 4, and 5 plot the residuals (differences) between the results of the fits and the experimental results, and table 1 lists the residual standard deviation for three different choices of *S* and *N*_ss_ It is clear by inspection of the figures that the combination of *S* and *N*_ss_ used for figure 3 gives a better overall fit than either of the other choices, even though the residual standard deviation for figure 3 is not very much lower than that for figure 4 or figure 5. One reason for the relatively small decrease in residual standard deviation with the best fit is the effect of the apparent outlier points near −1 and −10.5 *V* oxide bias.

The assignment of *S* = 6000 cm/s and *N*_ss_ = 1.942 × 10^12^ cm^−2^ with estimated uncertainties of ± 1000 cm/s and ± 0.070 × l0^12^ cm^−2^, respectively, is justified by figures 3, 4, and 5. This assignment corresponds to an internal quantum deficiency (one minus the internal quantum efficiency) at zero oxide bias of 0.1690 ± 0.0021. The stated uncertainty is the maximum difference between the internal quantum deficiency calculated for the combination of *S* and *N*_ss_ used in figure 3, and those calculated for the combinations of *S* and *N*_ss_ used for figures 4 and 5.

The assumption that the average of the last two data points in figure 3 corresponds to 100% internal quantum efficiency gives a zero-bias internal quantum deficiency of 0.1634 with no reliable way to estimate the uncertainty associated with that value. This assumption, which is usually used with the oxide-bias, self-calibration experiment, produces a result that differs from the more accurate value determined from the fit by 0.0056. This difference is about 3.3% of the effect being calculated, and more than 2.5 times the uncertainty associated with the more accurate value.

It must be emphasized that the results reported here are illustrative rather than definitive since the doping distribution data in figure 1 were obtained from one photodiode, and the oxide-bias data in figure 2 were obtained from a different photodiode. An additional uncertainty associated with the effect of variations in doping distribution would be needed, or the doping distribution must be measured on the actual photodiode used in the selfcalibration.

Because oxide-bias voltages large enough to nearly saturate the photodiode response are also large enough to change *S* or *N*_ss,_ the oxide-bias measurement is already destructive to a certain extent, and it must be carried out as described in reference [7] in order to yield the highest accuracy of which it is capable. Therefore, the sacrifice of the photodiode for a spreading resistance measurement is not as impractical as it might at first seem. Nevertheless, it is inconvenient, and more convenient approaches are being developed [3].

The results reported here might be considered illustrative for another reason. The fitting and uncertainty analysis were carried out by hand in a somewhat subjective manner. Ideally, the fitting and uncertainty analysis would be carried out automatically by a fitting routine incorporated into the set of programs used to run PC-1D for photodiode modeling. However, this task might not be as straightforward as it first would seem because the fit should be constrained to pass through the mean of the last few data points at the highest oxide-bias voltage, as it was in the hand fit reported here. Without this constraint, the minimum residual standard deviation might be obtained for a choice of parameters that clearly does not fit the data well at the highest oxide-bias voltages. It is clear that the data at the highest oxide-bias voltages should be weighted more heavily in the fit, but it is not clear how much more heavily. Therefore, an automated program does not eliminate the subjectivity, but only transfers it to the weighting of the data.

## 3. Hamamatsu 1337 Photodiode

**Figure 6:**
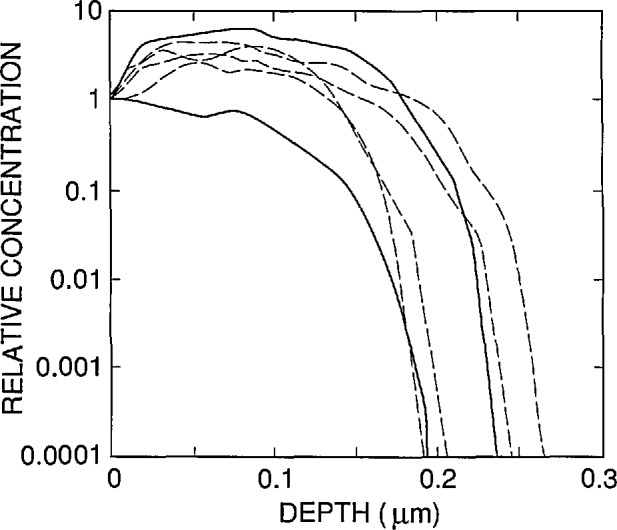
The relative front-region boron concentration for six typical Hamamatsu 1337 type photodiodes. The two profiles shown with solid lines bracket the other four in their effect on the shape of the internal quantum-deficiency spectrum.

Like the EG&G UV444B photodiode, the Hamamatsu 1337 is a *p ^+^nn* ^+^ photodiode, but its front-region doping profile is shallower, its background *n*-type dopant concentration is 5 × 10^12^ cm^−3^, and its oxide-passivation layer has a nominal thickness of 25 nm. Figure 6 shows relative front-region doping profiles for six different Hamamatsu 1337 photodiodes taken from four different batches. The curves in figure 6 were normalized to unity at the oxide-silicon interface because it is the shape of the doping profile rather than the magnitude that has the major effect on the internal quantum efficiency [8]. The measured dopant concentrations at the oxide-silicon interface varied between 2 × 10^18^ and 1 × 10^19^ cm^−3^ for the six photodiodes of figure 6.

**Figure 7:**
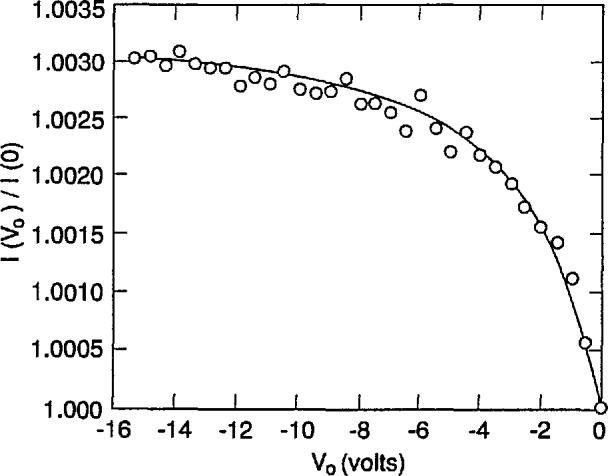
Ratio of the photocurrent *I*(*V*_0_) as a function of oxide-bias voltage *V*_0_ to the photocurrent *I*(0) at zero-bias voltage as measured on a Hamamatsu 1337 silicon photodiode and as calculated with PC-1D for the doping distribution corresponding to the upper solid curve in figure 6 with *S* = 1.835 × 10^5^ cm/s and *N*_ss_ = −3.0 × 10^12^ cm^−2^.

Figure 7 shows the results of modeling an oxide-bias experiment on a 1337 type photodiode at 476.2 nm by using the doping profile that corresponds to the lower solid curve in figure 6. The fit shown in figure 7 was obtained with *N*_ss_ = − 3.0 × 10^12^ cm^−2^ and *S* = 1.835 × 10^5^ cm/s, and gave a zero-bias internal quantum deficiency of 0.003199. A fit that looked virtually identical to that shown in figure 7 was obtained for the doping profile corresponding to the upper solid curve in figure 6 by using *N*_ss_ = −4 × l0^12^cm^−2^ and *S* = 1.56 × l0^5^ cm/s. This fit gave a zero-bias internal quantum efficiency of 0.003280. As in the case of the UV444B type photodiode, the SIL_WEAK.ABS absorption-coefficient data were used, and the rear of the photodiode was modeled as uniformly doped with zero recombination velocity.

**Figure 8:**
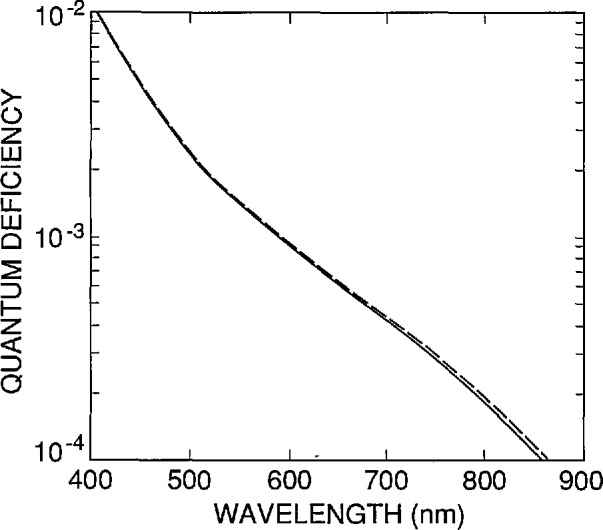
Simulated internal quantum deficiencies for the 1337 type photodiode having the oxide-bias data shown in figure 7: (solid curve) for the doping profile that corresponds to the lower solid curve in figure 6 with *N*_ss_ = −3.0 × 10^12^ cm^−2^ and *S* = 1.835 × 10^5^ cm/s, and (dashed curve) for the doping profile that corresponds to the upper solid curve in figure 6 with *N*_ss_ = −4 × 10^12^ cm^−2^ and *S* = 1.56 × 10^5^ cm/s.

Figure 8 compares the internal quantum deficiencies calculated from the doping profile corresponding to the solid curves in figure 6 by using the values of *N*_ss_ and *S* that were obtained from the simulated oxide-bias experiments based on each of those profiles. The fact that the two curves have very similar shapes is very important. It suggests that a single function of wavelength might be used to extrapolate the internal quantum efficiency of any 1337 type photodiode, rather than a different function for each different photodiode. This possibility is examined in detail in Part III of this series of papers.

Notice that the sign of the trapped charge in the 1337 type photodiode is the opposite of that for the UV444B photodiode, and that the interface recombination velocity is much larger. Both the sign of the trapped charge and the large value of the recombination velocity are consistent with an oxide prepared by chemical vapor deposition rather than by thermal oxidation, because the former is known to produce trapped positive charge and a low surface recombination velocity. The modeling carried out here shows that a large negative charge in the oxide more than makes up for the greater surface recombination velocity by repelling minority carriers from the surface.

The fact that the 1337 type photodiode has negative rather than positive charge trapped in its oxide is important for its possible use in quantum-efficiency interpolation and extrapolation. If the oxide contained a significant amount of positive charge, as assumed in reference [9], the oxide-silicon interface would be depleted of holes, leading to a very large decrease in the energy needed to impact-ionize a valence band electron. However, since the oxide contains negative charge, the interface is accumulated. In this case, even though the charge density is quite large, there is little or no decrease in the energy needed to impact-ionize a valence band electron [9]. This is important because a large decrease in the energy required to impact-ionize a valence band electron might cause the quantum yield [10] for electron-hole pair production of the silicon near the front surface to exceed unity at wavelengths as long as 450 nm. This would limit the usefulness of this type of photodiode for extrapolating and interpolating quantum-efficiency calibrations. If this limitation were encountered, it could only be overcome with a very accurate model of the quantum yield in the presence of surface fields. Such a model appears well beyond the current state of the art [9].

Even though the uncertainties in the internal quantum deficiency associated with the diode-to-diode variations in doping profile are quite small for the 1337 type photodiode, the oxide-bias experiment is still less than ideal for self-calibration [3]. However, since the 1337 is so well suited for interpolating and extrapolating internal quantum-deficiency data, as shown in Part III, all that is needed are ways to calibrate the photodiode that work over limited spectral regions near 450 nm and near 900 nm. The next section illustrates how simulation can be used to predict the internal quantum efficiency of a different type of photodiode with an uncertainty of about 0.0003 at 450 nm.

## 4. UDT UV100 Photodiode

The UDT UV100 type photodiode is usually assumed to have an internal quantum efficiency of unity in the short wavelength portion of the visible [11]. In fact, this assumption is the basis for the use of the QED 100 and QED 200 multiple reflection radiometers [12], which contain modified UV100 photodiodes, as high-accuracy absolute radiometric standards.

As the state of the art in absolute radiometry improves with time, the assumption of unity quantum efficiency for the UV100 type photodiode becomes more questionable. What is needed is a nominal internal quantum-deficiency spectrum accompanied by a reliable uncertainty spectrum. Since the 1337 type photodiode can be used to interpolate or extrapolate internal quantum-efficiency calibrations, as shown in Part III, it is only necessary to have these spectra over a limited spectral region above 400 nm. (Below 400 nm, the nonunity quantum yield would be a problem.) One way to obtain these spectra is to calculate them from nominal characteristics of the UV100 type of photodiode. The pertinent characteristics can be obtained by fitting numerical simulations of oxidebias measurements to experimental results as demonstrated in the preceding sections.

The UV100 type of photodiode is quite different from the UV444B and 1337 type photodiodes. Its *n ^+^p* junction is not formed by introducing a frontregion dopant, but is induced in a 100 Ω cm *p*-type (approximately 1.3 × l0^14^cm^−3^, boron-doped) silicon substrate by the growth of an oxide passivation layer having a large concentration of trapped positive charge [13].

**Figure 9:**
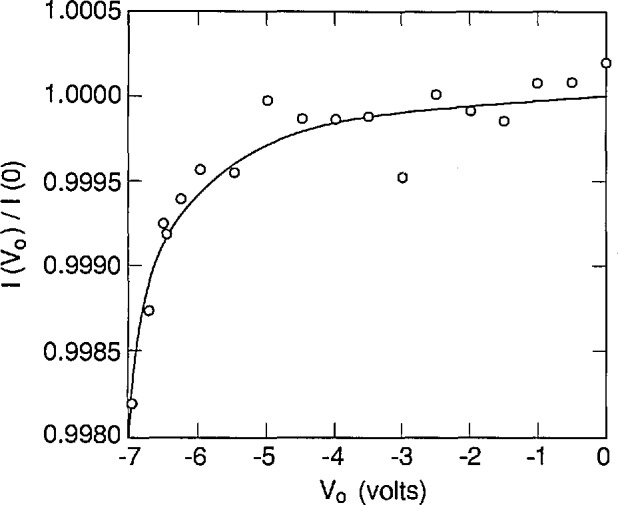
Ratio of the photocurrent *I*(*V*_0_) as a function of oxide-bias voltage *V*_0_ to the photocurrent *I*(0) at zero-bias voltage as measured on a typical UDT UV100 silicon photodiode and as calculated with PC-1D for 5 = 3.0 × 10^5^ cm/s and *N*_ss_ = 1.38 × l0^12^cm^−2^.

Figure 9 compares the results of simulated and experimental oxide-bias experiments on a UV100 type photodiode. Here again, the absorption-coefficient data in SIL_WEAK.ABS were used, and the rear of the photodiode was modeled as uniformly doped with a surface recombination velocity of zero. Since the UV100 type photodiode is an *n ^+^p* type rather than a *p ^+^n* type, the effect of increasing negative bias is to decrease rather than increase the photocurrent. However, because the zero-bias quantum efficiency is so close to unity, this procedure samples the portion of the curve with the most information about the values of *N*_ss_ and *S*, as can be seen in the figure.

**Figure 10:**
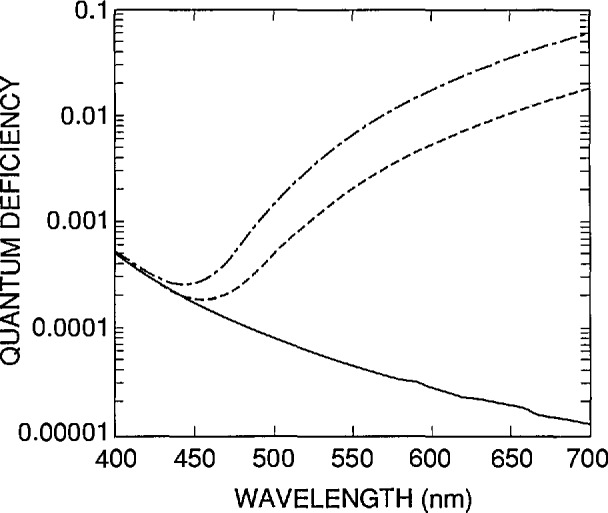
Internal quantum-deficiency spectra calculated by PC-1D for 5 = 3.0 × l0^5^ cm/s, *N*_ss_ = 1.38 × l0^12^ cm^−2^, and rearregion lifetimes τ of 81 ms (solid line), 10 µs (dashed line), and 1 µs (dot-dashed line).

These values of *N*_ss_ and *S* are used to simulate the internal quantum-deficiency spectra for a nominal UV100 photodiode in figure 10. The three different curves shown in figure 10 correspond to minority carrier lifetimes τ of 1 µs, 10 µs, and 81 ms, respectively, in the rear region of the photodiode. The lifetimes of 1 and 10 µs give internal quantum deficiencies at 700 nm that bracket the range measured for six typical UV100 type photodiodes [14], and the 81 ms lifetime is the longest lifetime that the default silicon material model of PC-1D will allow for 100 Ω cm, *p*-type silicon with a recombination trap at mid-gap.

**Figure 11:**
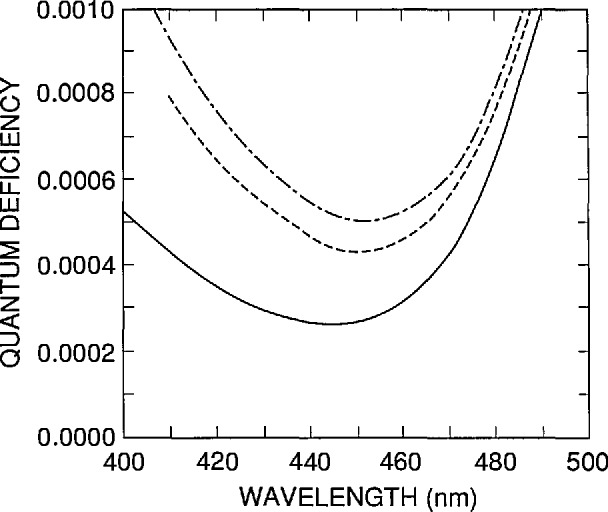
Internal quantum-deficiency spectra calculated by PC-1D for a rear-region lifetime τ = 1 µs with *S* =3.0 × 10^5^ cm/s and *N*_ss_ = 1.38 × l0^12^cm^−2^ (solid line), with τ = 1 μs with *S* =6.0 × 10^5^ cm/s and *N*_ss_ = 1.38 × 10^12^ cm^−2^ (dashed line), and with τ = 1 µs with *S* = 3.0 × 10^12^ cm/s and *N*_ss_ = 0.69 × l0^12^cm^−2^ (dot-dashed line).

The region between the curves corresponding to τ = 1 and τ = 10 µs in figure 10 is a nominal internal quantum-deficiency spectrum that applies to UV100 type photodiodes and to QED 100 and QED 200 radiometers at zero reverse bias [3,12]. Figure 11 compares the spectrum for τ = 1 µs around its minimum in the 400 to 500 nm spectral region with spectra obtained for the cases where either the front-region surface recombination velocity is doubled, or the positive charge trapped in the oxide is halved. These are conservative limits to associate with the mass production of photodiodes in a controlled planar-silicon fabrication process. These should also be conservative limits for the effects of environmental stress, such as humidity, temperature, and irradiation with ultraviolet radiation, for UV100 photodiodes [15–16] made after 1986.

The curves in figure 11 can be used to estimate uncertainties to be associated with the nominal values of the internal quantum efficiency of the UV100 type photodiode. Summing in quadrature the differences between the nominal spectrum and the other two spectra in figure 11 produces a somewhat conservative estimate since increases in surface recombination velocity tend to be correlated with increases in trapped charge. In the region from 440 to 460 nm, a nominal value of 0.0003 ± 0.0003 is a practical summary of the results of such an analysis. This result should be considered a one-standard-deviation equivalent, rather than a limit of error, since the whole analysis is based on the results obtained for a single device. Outside the 440 to 460 nm spectral region, the uncertainties grow so rapidly that they are not really practical for high-accuracy applications.

**Figure 12:**
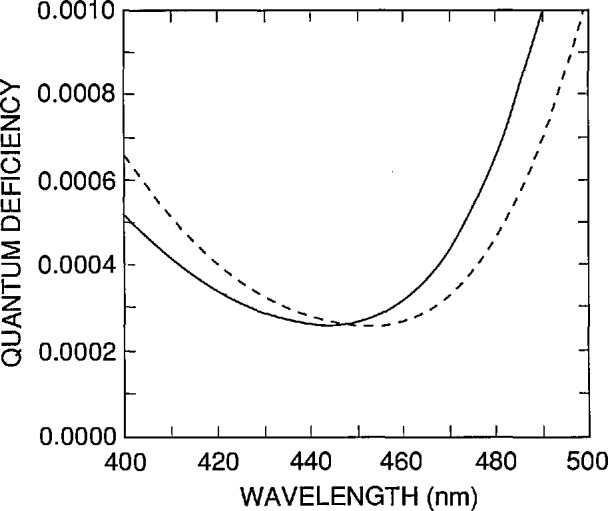
Comparison of the internal quantum-deficiency spectra calculated for a UV100 type photodiode for a rear-region lifetime τ = 1 μs with *S* = 3.0 × 10^5^ cm/s and *N*_ss_ = 1.38 × 10^12^ cm^−2^ with the absorption-coefficient data in the SIL_WEAK.ABS file (solid line), and with the absorption-coefficient data in the SIL-PHIL.ABS file (dashed line). The data in these files are described in more detail in Part I of this series of papers.

There are a number of sources of error not yet considered, but since their effect scales with the magnitude of the internal quantum deficiency, they are all negligible with respect to ± 0.0003. This is illustrated in figure 12 for the uncertainty associated with the absorption coefficient of silicon in the 440 to 460 nm spectral region, which is the largest uncertainty not considered so far.

Figure 12 compares the spectrum of figure 11 for τ = 1 μs, which was based on the absorption-coefficient data in SIL-WEAK.ABS, with the same spectra calculated for identical parameters, but based on absorption-coefficient data (measured by Philipp [17], and described in Part I) that is stored in SIL-PHIL.ABS. When added in quadrature to the ± 0.0003 assigned to the variations in *N*_ss_ and *S* from diode to diode, the effect of the uncertainty in the absorption coefficient is negligible. This might not remain true if a much larger wavelength interval were considered.

Because of the large long-wavelength quantum deficiency shown in figure 10, reverse bias is often applied to UV100 photodiodes and QED 100 and 200 radiometers when they are used at the longer wavelengths. By moving the depletion region toward the rear of the photodiode, the reverse bias forces the internal quantum deficiency to approach the lower quantum deficiency limit given by the 81 ms curve in figure 10 [12,18–19]. Reverse bias is also applied to eliminate the saturation type non-linearity that is sometimes observed with photocurrents above a few microamperes. However, recent results [4] suggest that moderate bias levels can cause gain, resulting in an internal quantum efficiency in excess of unity. This effectively precludes the application of moderate reverse bias in high-accuracy applications.

**Figure 13:**
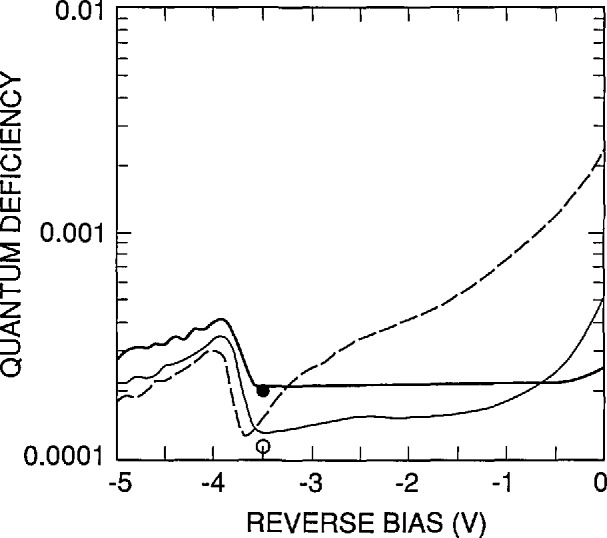
Simulation of the decrease in internal quantum-deficiency of a UV100 type photodiode with reverse bias at 442 nm (heavy solid line), 476 nm (light solid line), and 514 nm (dashed line). The values of *N*_ss,_ *S*, and τ are as in figure 12. The feature near −3.5 V is an artifact of the simulation. The open circle and closed circle are the internal quantum deficiencies at 442 nm and 476 nm, respectively, for τ = 81 ms. The internal quantum deficiency at 514 nm for the same condition is too small to appear on the graph.

Figure 13 plots a simulation of the decrease in the internal quantum deficiency as a function of reverse bias for the UV100 type photodiode at the 442, 476, and 514 nm laser lines. The feature in the curves for reverse-bias voltages less (more negative) than −3.5 V is a symptom of a problem with PC-1D. It may mean that more than 150 finite elements are needed to maintain the level of accuracy achieved as the reverse bias is increased, but this is not certain. In any case, it not only invalidates the data for reverse-bias voltages more negative than −3.5 V, but it also casts some doubt on any conclusions drawn about the data at the less negative bias voltages as well. At best, figure 13 suggests that 480 nm is a practical upper limit to the wavelength at which reverse bias can be used to eliminate the effect of recombination in the rear region of the photodiode without risking gain. However, the best advice would be never to use a UV100 photodiode or QED radiometer with a bias voltage without first assuring experimentally that gain is not a problem.

## 5. Conclusion

Part II of this series of papers has demonstrated the use of Version 2 of PC-1D.EXE, RUN_PC1D.BAT, MAKE_PRM.EXE, and READ_PDF.EXE to simulate various oxide-bias experiments of interest to high-accuracy applications of silicon photodiodes. In each case, the simulation was used to interpret an oxide-bias experiment, with different sorts of information being sought from the different experiments.

It was shown that the simulations can be used to derive a more accurate internal quantum-deficiency value from an oxide-bias experiment than that available from the conventional data reduction, and that an uncertainty can be associated with the value derived from the simulation based on how well the simulated oxide-bias data can be fitted to the experimental data. It was also shown that simulations can be used to determine nominal values for the front-surface recombination velocity and for the charge trapped in the front-surface oxide, and that values for these parameters can be used to determine nominal internal quantum-deficiency spectra for various types of silicon photodiodes.

As well as illustrating these general ideas, some more specific results were also obtained. First, it was shown that a nominal internal quantum deficiency of 0.0003 ± 0.0003 is appropriate for the unbiased UDT UV100 type photodiode in the 440 to 460 nm spectral region. Second, it was shown that the shape of the internal quantum-deficiency spectrum of the 1337 type photodiode is quite independent of typical variations in doping profile. This result suggests that the 1337 type photodiode might serve to extrapolate high-accuracy calibrations obtained from cryogenic absolute radiometers or from QED type radiometers in the 440 to 460 nm spectral region to longer wavelengths with little or no loss of accuracy. Part III of this series of papers shows that this is the case. Finally, it was shown that reverse-bias simulations based on the programs described in Part I of this series of papers do not work well enough with inversion layer (induced junction) photodiodes to be useful for high-accuracy applications.

## Figures and Tables

**Table 1:** Comparison of simulated oxide bias results with experimental results for a UV444B type photodiode where Res. std. dev. is the standard deviation of the difference between simulated and experimental results

*S* (cm/s)	*N*_ss_ (10^12^ cm^−3^)	Res. std. dev.
5000	2.011	0.002233
6000	1.942	0.002076
7000	1.883	0.002093

## Footnotes

1 Certain commercial equipment, instruments, or materials are identified in this paper to specify adequately the experimental procedure. Such identification does not imply recommendation or endorsement by the National Institute of Standard and Technology, nor does it imply that the materials or equipment identified arc necessarily the best available for the purpose.
